# Quantitative and chemical adaptation of exopolymeric substances formed by a river microbial consortium during exposure to the antibiotic trimethoprim

**DOI:** 10.1016/j.bioflm.2025.100334

**Published:** 2025-11-19

**Authors:** Thibault Duteil, Andras Gorzsás, Madeleine Ramstedt

**Affiliations:** Department of Chemistry, Umeå University, 90187, Umeå, Sweden

## Abstract

In natural environments, most microorganisms reside attached to a surface growing as a biofilm, which is a universal microbial strategy for their survival. As a result, several studies have focused on the role of the biofilm matrix (made of extracellular polymeric substances (EPS)) in tolerance to antimicrobials. However, few studies have focused directly on the characterization of EPS as a response to antibiotic stress. In this study, we analyzed the impact of trimethoprim (TMP) on the production and characteristics of EPS from four natural biofilm-producing river bacterial strains. Extraction and characterization of EPS were carried out at three concentrations of TMP. EPS properties were monitored using colorimetric tests, infrared spectroscopy and sugar composition analysis. For all strains, EPS quantity and chemistry changed starting at 0.1 mM TMP. The combined results suggest that environmental bacterial strains adapt their EPS production and chemical composition in response to antibiotic exposure. Bacteria may benefit from the change in EPS chemistry since it limits the penetration of TMP into the biofilm and thus protects the cells from the action of the antibiotic. Three main mechanisms are proposed: an increase in the proportion of (i) proteins and reactive functional groups, (ii) mannose and (iii) fatty acids. This study shows that EPS represents a key factor in antibiotic tolerance of bacteria via multiple mechanisms. Thus, this study broadens the discussion concerning antibiotic-resistance as it presents additional processes that may work in tune with genetically acquired resistance to enhance bacterial tolerance to antibiotics.

## 1 Introduction

Pharmaceuticals are an emerging problem in various ecosystems because they retain their biological activity also when they are released into the aquatic environment [1,2]. Initially designed to help the human body, pharmaceuticals can also interact with other organisms in the environment. Biological effects of pharmaceuticals occur in the μg·L^−1^ and ng·L^−1^ concentration ranges [87] (Richardson & Ternes, 2021) resulting in an environmental stress factor for organisms that may lead to changes in behavior, metabolism and/or survival. A prime example are antibiotics that can affect prokaryotic cells by various specific mechanisms of action e.g., inhibition of protein synthesis or disruption of their metabolism [3]. For example, trimethoprim (TMP), an antibiotic commonly used for treating urinary tract infections [4], induces toxic effects on green alga and cyanobacteria [5]. TMP inhibits the function of dihydrofolate reductase and, as a result, blocks nucleic acid and protein synthesis in bacterial strains [6]. TMP concentration in European rivers is predicted to be around 0–0.06 μg L^−1^ with the worst case around 0–0.23 μg L^−1^ [7].

In natural environments, most microorganisms reside attached to a surface growing as a biofilm, which is a universal microbial strategy for their survival [8]. A biofilm is an organized aggregate of microorganisms living within a self-produced matrix of extracellular polymeric substances (EPS) attached to a surface. About 90 % of biofilm biomass is comprised of the EPS matrix [9]. This matrix consists of a mix of proteins, polysaccharides, lipids, nucleic acids, extracellular DNA, phospholipids and other organic compounds [10]. EPS plays several important roles for the microbial community within the biofilm, such as adhesion to the substrate, aggregation of cells, water retention, trapping of molecules (e.g., organic compounds or inorganic ions), nutrient resource, enabling information exchange within the matrix, as well as serving as a protective barrier against pollutants and antimicrobials [11]. As a result, biofilm cells are less sensitive to the effects of antimicrobial agents, including antibiotics [12]. Ceri et al [13] compared the antibiotic sensitivity of planktonic populations and biofilms of the same organisms and showed that a concentration 100 to 1000 times greater was often required for an antibiotic to be effective in the biofilm. Two mechanisms may explain antibiotic tolerance in the biofilm: (i) mechanisms that prevent the antibiotic from accessing its target and (ii) mechanisms that actively remove the antibiotic from the cell via degradation or efflux pumps [14].

The EPS matrix plays an important role, preventing antibiotics from reaching cells, transforming them, and enabling antibiotic resistance gene transfers [15,16]. The importance of decreased antibiotic penetration depends on several variables (including bacterial strain and antimicrobial agent) and it has been proposed that slower penetration may give time for an adaptive phenotypic response increasing tolerance [17]. The main process of this mechanism is electrostatic interactions by polysaccharides that physically sequester antibiotics in the biofilm EPS [18,19]. This may work together with genetically encoded resistance mechanisms for example, via synthesis of antimicrobial efflux pumps, moving antimicrobial agents away from their intracellular targets and back out into the extracellular space, conferring resistance to cells [20]. The EPS matrix can also act as an accumulation space for antibiotic-degrading enzymes, reducing the quantity of antibiotics by hydrolysis [21].

Previous studies have focused on the role of EPS in tolerance to antimicrobials. For example, the impact of different polysaccharide species (e.g., Pel; [22,23]) or the quantity of EPS produced as a function of environmental factors [24]. However, few studies have focused directly on the production and physico-chemical composition of EPS as a response to antibiotic stress. In this study, we analyzed the impact of TMP on the production and characteristics of EPS from four natural river bacterial strains. These bacteria produce EPS and have the ability to form biofilms ([25]., in review). The isolates came from a river affected by discharges from sewage treatment plant. Thus, they had been exposed to low levels of pharmaceuticals (including TMP) prior to isolation and may have adapted their lifestyle to this type of stress. The work by Ref. [25]. (under review) has shown that the isolates are affected by the presence of TMP as a stress factor, which is why this antibiotic was selected to test its potential impacts on EPS production and characteristics. Extraction and characterization of EPS were carried out at three concentrations of TMP. Physico-chemical properties of EPS were monitored using colorimetric tests, infrared spectroscopy and sugar composition analysis. Scanning electron microscopy observations were made to see potential changes in the organization of the biofilm in the presence of trimethoprim. The aim of this study was to determine the impact of antibiotics on the production and/or chemical modification of EPS for four environmental river bacterial strains.

## 2 Method

### 2.1 Growth condition

**Fig. 1:**
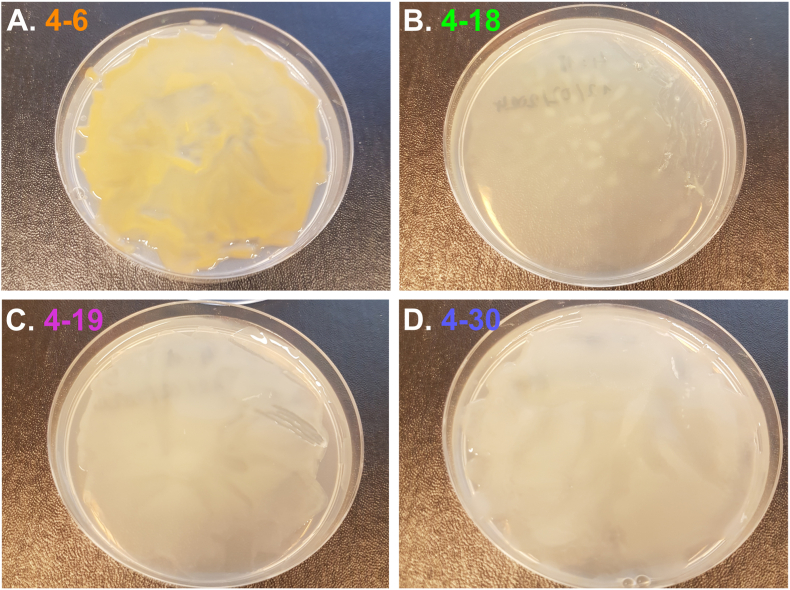
Picture of a lawn of bacteria from each strain used in this study after three days of growth on agar plates before EPS extraction and purification: (A) 4–6 *Sphingomonas s.p.*, (B) 4–18 *Pseudomonas s.p.*, (C) 4–19 *Rhizobium s.p.* and (D) 4–30 *Pararhizobium s.p.*

Four previously well-characterized river isolates were cultivated in 100 % R2A medium agar plates (Sigma-Aldrich; [26]): 4–6 *Sphingomonas*, 4–18 *Pseudomonas*, 4–19 *Rhizobium* and 4–30 *Pararhizobium s.p.* ([25]., in review). Bacterial isolates came from Knivstaån stream, in the south of Sweden (Latitude: 59° 43′ 32.30″ N, Longitude: 17° 47′ 15.11″ E). The isolates were collected in May 2018, downstream from Knivsta town wastewater treatment plant. All four came from the same sampling point (sampling point 4 described in Ref. [27]). The overall bacterial flora of the sampling points is described in Ref. [27]. Each strain produced biofilm (Fig. 1) and are available at the Göteborg university culture collection (4:06 - CCUG 78359; 4:18 - CCUG 78360; 4:19 - CCUG 78361; 4:30 - CCUG 78362). Concentration of TMP in the Knivstaån stream water ranged from 0,07 to 0,34 mM from May to October 2018 (0.02–0.1 μg L^−1^; [27]). Based on these values, 0.1 mM was chosen to be a stress-threshold test quite close to a realistic chronic exposure in the natural environment. 10 plates of each strain were cultivated at room temperature (21ᵒC) for five experimental conditions: no solution addition, addition of 50 μl of 1 % acetic solution (pH: 3.00), addition of 50 μl of 0.1 (pH: 3.39), 1 (pH: 3.05) and 5 mM (pH: 3.43) trimethoprim in 1 % acetic solution. Please note that the pH values given are for the separate solutions added to the agar plates. TMP solutions were made on 1 % acetic acid solution to increase its solubility [28]. Acetic acid solution was used as a control. A total of three days of growth were performed for all experimental conditions. For the relevant experimental conditions, after one day of growth, 0.5 mL of TMP or acid solution was added to completely cover the bacterial biofilm (Table 1). The bacteria then continued to grow for two days, reaching a total of three days of growth. After three days EPS was extracted by gently recovering all the organic material on the surface of the 10 agar plates by the use of culture loops. Care was taken not to include any agar from the R2A plates. Experiments were carried out on 50 plates (10 for each experimental condition) for each of the four strains making a total of 200 plates.

**Fig. 2:**
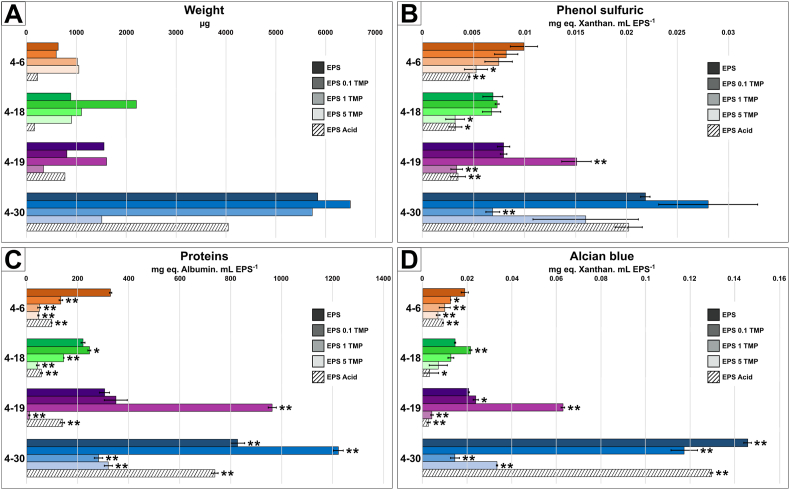
Results of EPS extraction and characterization with colorimetric assays. Five experimental growth conditions with increasing concentrations were applied before EPS extraction. In the bar plot bacterial strains are color coded: 4–6 *Sphingomonas s.p.* (orange), 4–18 *Pseudomonas s.p.* (green), 4–19 *Rhizobium s.p.* (purple) and 4–30 *Pararhizobium s.p.* (blue). The color intensity of the bar in the plot corresponds to stepwise increased concentrations of trimethoprim (TMP): from no addition (darkest color), 0.1 mM of trimethoprim (medium dark color), 1 mM of trimethoprim (medium light color), and 5 mM of trimethoprim (lightest color). Acidic control is represented by dashed bars. Total dry weight of EPS (A), total concentration (mg·mL^−1^) of sugar based on the Phenol sulfuric assay (B), amount of protein based on the modified Lowry assay (C), and reactivity based on the quantity of negatively charged reactive sites in EPS capturing the Alcian blue stain, from method by Passow and Alldredge (D). Data represents averages of three replicates; error bars represent standard deviation. Statistical significance: ns > 0.05, ∗p ≤ 0.05, ∗∗p ≤ 0.01.

**Table 1:** Experimental growth conditions before EPS extraction.

Sample name	Solution addition after 1 day of growth in R2A plate
EPS	None
EPS Acid	50 μL of 1 % acetic acid
EPS 0.1T	50 μL of 0.1 mM trimethoprim in 1 % acetic acid
EPS 1T	50 μL of 1 mM trimethoprim in 1 % acetic acid
EPS 5T	50 μL of 5 mM trimethoprim in 1 % acetic acid

### 2.2 EPS extraction

For EPS extraction, the method used was developed by Underwood et al. [29]. It consists of DI water extraction followed by cold propanol precipitation. This method ensures good extraction efficiency without changing the physical properties of proteins or polysaccharides within the EPS [30,31]. For all strains, EPS was extracted by diluting the biofilm collected from plates with two volumes of deionized (DI) water. The solution formed was put into an ultrasonic bath for 10 min and thereafter gently stirred for 10 min. Centrifugation was performed (4200×*g*, 20 min) to remove cells. The recovered supernatant was filtered through polycarbonate filters (0.22 μm). Propanol was stored beforehand in the freezer to cool it down. The filtrate was precipitated using three volumes of cold propanol (<0 °C) per volume of filtrate and left for a day in the freezer. The precipitate was recovered by centrifugation (4200×*g*, 10 min), placed in dialysis tubing (10 kDa), dialyzed first for two days in a buffer solution (2 l of DI water at 1 mm EDTA) and then two more days against 2 l of DI water (>18 MΩ). Liquid EPS solutions were stored in the refrigerator at 4 °C until analysis.

### 2.3 Colorimetric assays

The composition of EPS was estimated using three colorimetric assays: (i) phenol-sulfuric acid assay [32], (ii) Alcian Blue assay [33], and (iii) Pierce-modified Lowry assay [34]. All these assays are based on the Beer-lambert law for the calculation of the concentration of colored complexes or molecules formed by the reaction. The phenol-sulfuric acid assay is used to measure the concentration of sugar polymers in EPS. Pure sulfuric acid hydrolyzes the sugar polymers and phenol reacts with the formed monomers, resulting in a dark brown colored compound. This provides an estimate of (i) the amount of sugars in EPS. The Alcian Blue method is based on the reaction of positively charged Alcian Blue with negatively charged reactive functions on EPS (mainly carboxylic acids and sulfates). Through the fixation on these sites, the assay is a proxy for (ii) the density of negatively charged reactive functions in the EPS. The Pierce modified Lowry assay consists in the reaction of proteins with cupric sulfate and tartrate in alkaline solution, resulting in the formation of tetradentate chelated copper complexes. This complex produces a blue color measurable at 750 nm, related to (iii) the amount of proteins. The standards were xanthan for the phenol-sulfuric and Alcian Blue assays, and bovine serum albumin for the Pierce-modified Lowry assay. Triplicates (100 μl) were used for each assay with the error bars in the figures. Absorptions were measured on a UV–visible Spectrometer Lambda 750 (PerkinElmer, USA). All remaining EPS were freeze-dried and weighed for each experimental condition. Detailed protocols are available here: [35].

### 2.4 Fourier Transform Infrared spectroscopy (FTIR)

Fourier Transform Infrared Spectroscopy analyses of the EPS samples were performed on a Vertex 80v (Bruker Optik GmbH, Ettlingen, Germany) Spectrometer in Attenuated Total Reflection (ATR) mode, using Bruker's own Platinum accessory with a diamond internal reflection element (IRE). One drop of EPS solution was placed on the diamond internal reflection element and evaporated under vacuum conditions. This was repeated three times to concentrate EPS on the diamond surface to increase the signal. Spectra were acquired in absorbance over the range 400 to 4000 cm^−1^ with a spectral resolution of 4 cm^−1^. Prior to sample measurements, the background was recorded on the clean diamond IRE and used for automatic background subtraction in all spectra, using Bruker's OPUS software (version 6.5). Each spectrum corresponds to a co-addition of 200 scans. To compare the results, spectra were exported to ASCII data files and processed by an open source, in-house Matlab (Mathworks Inc, USA) script (https://www.umu.se/en/research/infrastructure/visp/downloads/), based on the protocol by Felten et al. [36], using the following parameters: asymmetrical least squares baseline correction (lambda = 1 × 10^9^ and p = 0.001) and Total Area Normalisation over the 400-4000 cm^−1^ spectral range.

### 2.5 Scanning Electron Microscopy (SEM)

For the four strains, two different experimental conditions were observed with SEM: non-exposed and exposed to 1 mM TMP. After three days of growth, a small amount of the biofilm was collected from the surface of the R2A agar plates and deposited in poly-l-lysine coated coverslips. Directly after, to avoid disruptions or cell collapse, samples were fixed and dehydrated through a series of ethanol baths following the Malyshev et al. [37] method. Thereafter, samples were sputter coated with 5 nm of Iridium using a Quorum Q150T-ES (Quorum technologies, UK). Samples were analyzed on a Carl Zeiss Merlin FESEM (Zeiss, Germany). *JMicroVision* software was used to measure cell size and the percentage of area covered by EPS [38]. Ten cells were randomly selected from each SEM image.

### 2.6 Trimethylsilyl (TMS)-derivatization and GC/MS analysis of monosugar residues

The determination of total monosaccharide residues in EPS was performed according to the method by Ref. [39]. Extracted EPS was freeze-dried and approximately 500 ± 30 μg of each sample was weighted up in triplicates. 30 μg inositol was added to each sample as internal standard. Monosaccharide standards consisting of arabinose, rhamnose, fucose, xylose, glucuronic acid, galacturonic acid, mannose, glucose and galactose (Merck KGaA, Darmstadt, Germany) were prepared in a similar way. Sugar quantification was done by gas chromatography–mass spectrometry (GC-MS 7890A/5975C; Agilent Technologies, Santa Clara, U.S.).

### 2.7 Statistical analyses

The statistical analyses were performed using the R software [40]. Correlation matrices were calculated with the cor function and visualized with the package *Corrplot*. *rcorr function* of the package *Hmisc* was used to calculate the level of significance for the Pearson and Spearman correlations.

## 3 Results

To make it easier to read the results, each bacterial strain is assigned to a color in all Fig. 4, Fig. 5, Fig. 6 *Sphingomonas s.p.* (orange), 4–18 *Pseudomonas s.p.* (green), 4–19 *Rhizobium s.p.* (purple) and 4–30 *Pararhizobium s.p.* (blue). Tables with the data used in this study are available as supplemental information (Tables S1 and S2).

**Fig. 3:**
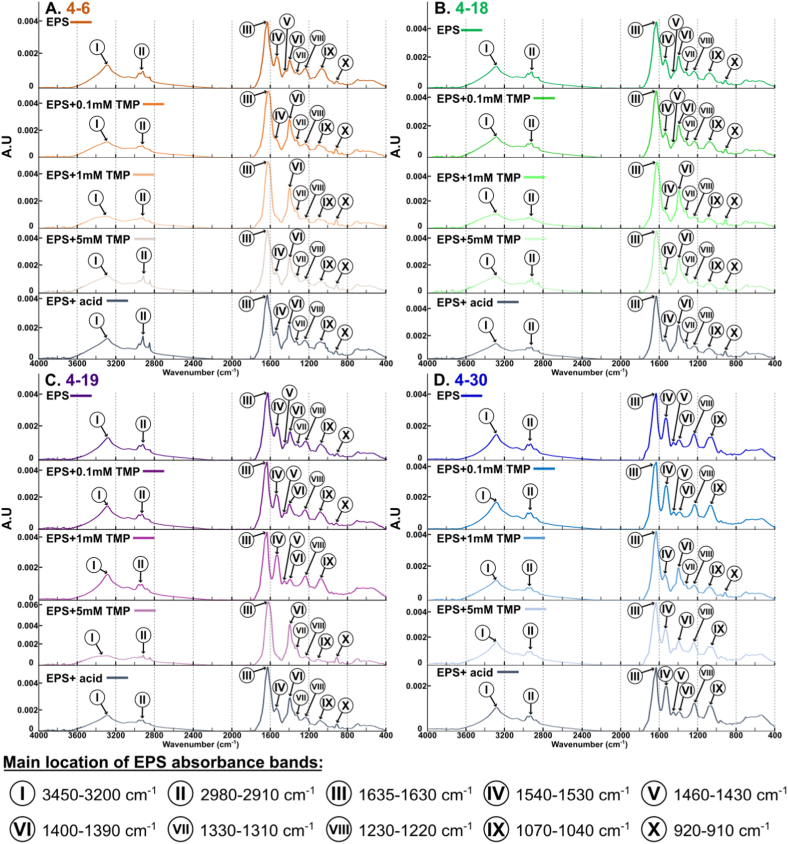
Fourier Transform Infrared (FTIR) spectra of purified EPS. Five experimental growth conditions were applied to bacterial cultures before EPS extraction: pure medium (labelled EPS in figure), 0.1 mM of trimethoprim, 1 mM of trimethoprim, 5 mM of trimethoprim and acidic control. Bacterial strains are shown in: (A) 4–6 *Sphingomonas s.p.* (orange), (B) 4–18 *Pseudomonas s.p.* (green), (C) 4–19 *Rhizobium s.p.* (purple) and (D) 4–30 *Pararhizobium s.p.* (blue). Main absorbance bands are marked by Roman numbers, with their positions listed at the bottom as ranges in wavenumbers.

**Fig. 4:**
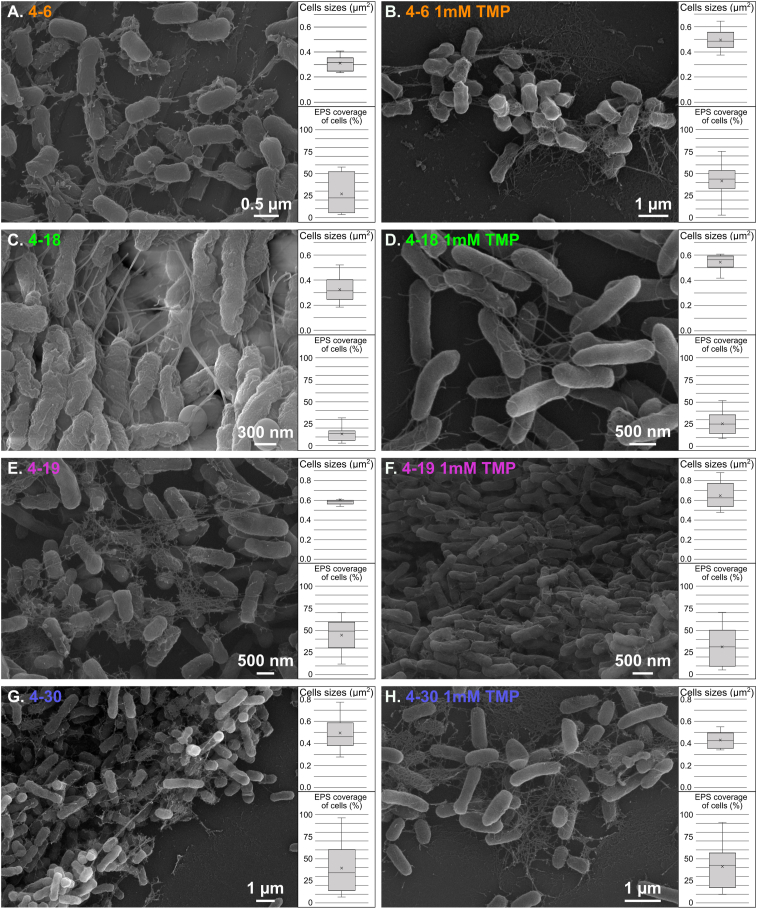
Scanning Electron Microscopy (SEM) images. Two experimental growth conditions are shown: pure medium (left panel, i.e., A, C, E, G) and 1 mM of trimethoprim addition (right panel, i.e., B, D, F, H). Bacterial strains are shown in A, B: 4–6 *Sphingomonas s.p.* (orange), C, D: 4–18 *Pseudomonas s.p.* (green), E, F: 4–19 *Rhizobium s.p.* (purple) and G, H: 4–30 *Pararhizobium s.p.* (blue). All the images show the presence of a large quantity of EPS dehydrated and collapsed into filamentous structures binding the cells together. On the right part of each image, boxplots of cell surface and percentage area of cells covered by EPS, measured on ten randomly selected cells.

**Fig. 5:**
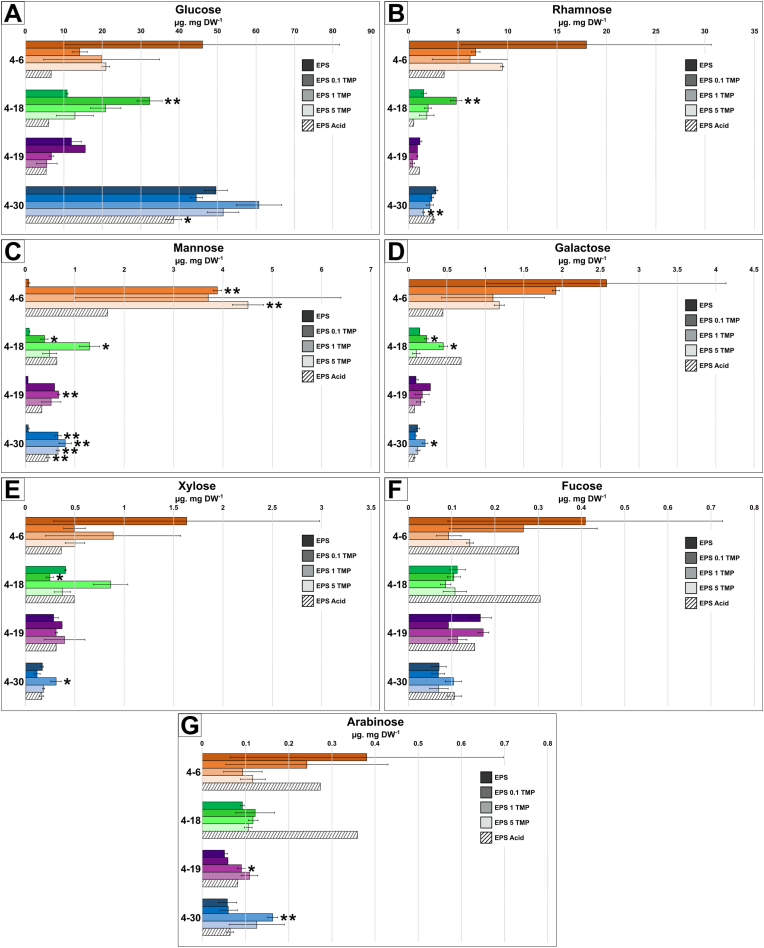
Monosaccharide composition of EPS: (A) glucose, (B) rhamnose, (C) mannose, (D) galactose, (E) xylose, (F) fucose and (G) arabinose. Five experimental growth conditions were applied before EPS extraction. In the bar plot, bacterial strains are color coded: 4–6 *Sphingomonas s.p.* (orange), 4–18 *Pseudomonas s.p.* (green), 4–19 *Rhizobium s.p.* (purple) and 4–30 *Pararhizobium s.p.* (blue). The color intensity of the bar in the plot corresponds stepwise to increased concentration of trimethoprim (TMP): pure medium (darkest color), 0.1 mM of trimethoprim (medium dark color), 1 mM of trimethoprim (medium light color), 5 mM of trimethoprim (lightest color). Acidic control is represented by dashed bars. Data represents averages of three replicates; error bars represent standard deviation. Statistical significance: ns > 0.05, ∗p ≤ 0.05 and ∗∗p ≤ 0.01.

**Fig. 6:**
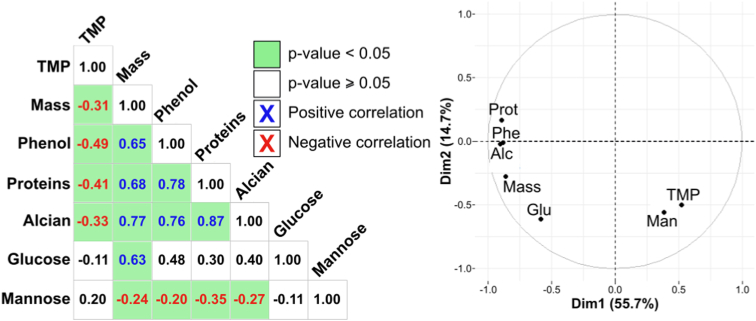
Principal Components Analysis (PCA) of selected data used in this study. Left part, correlation matrix of Pearson's r coefficients. Green boxes indicate a p-value <0.05. Right part, PCA correlation circle plot.

### 3.1 Extraction and physicochemical properties of EPS

The results of the EPS characterization are shown in Fig. 2. Weight results correspond to the total dry mass of the extracted EPS for each experimental condition. As a result, there is only one measure for each experimental condition, hence the lack of error bars. Strain 4-6 produced the least amount of EPS (633 μg; Fig. 2A), followed by 4–18 (891 μg; +41 %), 4–19 (1556 μg; +146 %), and 4–30 which is the most productive (5840 μg; +823 %). Results of the assays are expressed as milligrams of standard per milliliter of EPS. Standards are relative to albumin (protein assay) or xanthan (phenol-sulfuric and Alcian Blue assays). Error bars correspond to the standard deviation between triplicates for each assay. For clarity, all the strains will be discussed separately: For *Sphingomonas s.p.* (4–6, orange; Fig. 2), the mass of extracted EPS increased in the presence of TMP from 633 to 1044 μg for 5 mM TMP (Fig. 2). The acid control resulted in lower EPS production (220 μg). In the assays, an increase in TMP concentration resulted in a decrease in sugars ((9.9 ± 0.1)·10^−3^ to (5.3 ± 0.1)·10^−3^ mg eq. Xanthan·mL EPS^−1^), proteins (329 ± 4 to 46 ± 2 mg eq. Albumin·mL EPS^−1^) and EPS negatively charged reactive sites ((1.9 ± 0.01)·10^−2^ to (6.9 ± 0.08)·10^−3^ mg eq. Xanthan·mL EPS^−1^). For *Pseudomonas s.p.* (4–18, green; Fig. 2), the mass of extracted EPS remained quite stable in the presence of TMP (c. 965 μg) except for 0.1 mM TMP with a higher amount of EPS extracted (2207 μg). Again, the acid control resulted in lower EPS production (160 μg). For the sugars, the amount remained quite stable around (7 ± 0.9)·10^−3^ mg eq. Xanthan·mL EPS^−1^ from 0 to 1 mM TMP concentration then decreased to (3.2 ± 0.9)·10^−3^ mg eq. Xanthan·mL EPS^−1^ for 5 mM TMP. The amount of protein decreased from 221 ± 7 to 43 ± 5 mg eq·Albumin·mL EPS^−1^ in relation to TMP increase. The same observation was made for the amount of negatively charged reactive sites, except for the 0.1 mM TMP sample ((2.2 ± 0.05)·10^−2^ mg eq. Xanthan·mL EPS^−1^). For *Rhizobium s.p.* (4–19, purple; Fig. 2), the mass of extracted EPS did not seem to follow a clear pattern according to TMP concentration. Nevertheless, the least mass of extracted EPS was for 5 mM TMP. For the three assays, the TMP increase was linked to higher amounts until 5 mM TMP, which led to a sharp drop. Finally, for *Pararhizobium s.p.* (4–30, blue; Fig. 2), the mass of extracted EPS was large (c. 6000 μg) compared to other strains, except at 5 mM TMP, which resulted in a four-fold decrease compared to non-exposed biofilms. The extracted EPS contained a significant amount of sugars, proteins and negatively charged reactive sites for 0 and 0.1 mM TMP. However, a drop was observed when the TMP concentration exceeded 1 mM.

### 3.2 Fourier transform infrared spectroscopy

EPS extracted from the cultures displayed several major infrared absorption peaks typical of polysaccharides, proteins, and also of nucleic acids and lipids with carboxyl, hydroxyl and amino groups as the main functional groups ([41]; Fig. 3): (I) The very broad peak around 3250 corresponded to O–H stretching; (II) the vibrational bands in the spectral range 2980–2910 cm^−1^ represented the symmetric and antisymmetric stretching of C–H from CH_2_ and CH_3_ groups; (III) the peak between 1635 and 1630 cm^−1^ was amide I with a high contribution of C=O stretching; (IV) the peak between 1540 and 1530 cm^−1^ was amide II (high contributions of C–N stretching and NH bending of in the peptide bond); (V) the vibrational bands in the spectral range 1460–1430 cm^−1^ represented CH_2_ scissoring vibrations; (VI) peaks around 1400-1390 cm^−1^ were likely due to C-OO^−^ stretching potentially associated to amino acids; (VII) 1330-1310 cm^−1^ peaks were difficult to identify but could probably correspond to CH_3_ or C=O groups; (VIII) vibrational bands in the spectral range 1230–1220 cm^−1^ were assigned to C–N stretch and associated with secondary amides of proteins (amide III); (IX) 1070-1040 cm^−1^ region corresponded to vibrations of C–OH and C–C bonds in polysaccharides and alcohols; and (X) the peak around 920-910 cm^−1^ was hard to characterize but could potentially originate from HC=CH stretching or the C–O–C part of glycogen. The differences in the extracted EPS spectra between the strains only concerned the intensity of the peaks and some minor shifts. Thus, the bacteria were broadly similar in terms of EPS composition. Interestingly, increasing the concentration of TMP led to some modifications of the EPS chemical composition: the intensities of peaks (III) between 1635 and 1630 cm^−1^ and (VI) around 1400-1390 cm^−1^ increased. On the other hand, peaks (IV) around 1545-1530 cm^−1^ and (V) between 1430 and 1420 cm^−1^ disappeared, while the intensities of the peaks around (I) 3260, (II) 3080–2850 and (VII) 1225-1220 cm^−1^ remained mostly unchanged. Changes occurred from 1 mM TMP for 4–6, 4–18 and 4–30 strains. For the 4–19 Rhizobium s.p., this happened when the TMP concentration reached 5 mM.

### 3.3 Scanning electron microscopy

SEM images revealed that all the strains secreted EPS, observed as structures resembling fine alveolar or filamentous strands surrounding the intact cells (Fig. 4). In agreement with visual appearance on culture plates (Fig. 1), SEM images showed that some strains secreted more EPS than others, with 4–30 being the most productive (Fig. 4G and H). Cells sizes agreed with previous work ([25]., in review). SEM observations did not show any visual impact of TMP on the cell shape or on the production or shape of EPS, i.e., images appeared to have similar morphology in the presence and absence of 1 mM TMP (Fig. 4). Analysis of the images using JMicroVision software showed that cell size remains quite stable in the presence or absence of TMP as well as EPS coverage percentage (Fig. 4 and Table S3).

### 3.4 Sugar composition

Regarding the EPS monosaccharide profiles, all mono-sugar hydrolysates largely consisted of glucose (around 65–85 % of the total monosaccharides dry-weight) and rhamnose (around 5–25 % DW; Fig. 5) for all the strains. Interestingly, the presence of the TMP led to an increase in the amount of mannose for all strains (100-fold for the 4–6, 2–5 fold for the 4–18, 10–20 fold for the 4–19 and 10 fold for the 4–30; Fig. 5C). For other mono-sugars, the trend seemed less clear. For the 4–6 strain, high TMP concentration caused a decrease in the amount of all mono-sugars except galactose. The 4–18 strain produced more glucose and galactose in the presence of TMP with no real change for other sugars. For the 4–19 strain, TMP led to glucose decrease and arabinose and galactose increase. Finally, for the 4–30 strain, no stark differences were noticed except for a small increase in arabinose at high TMP concentration.

### 3.5 Statistical analysis

The first axis of the PCA explained 55.7 % of the variation and the second axis accounted for 14.7 % of the variation (Fig. 6). The correlation analyses revealed three groups of variables: (i) TMP and mannose in the positive part of axe 1, (ii) mass and glucose in the negative part of both axes and (iii) colorimetric assays result on the positive part of axis 2.

## 4 Discussion

**Fig. 7:**
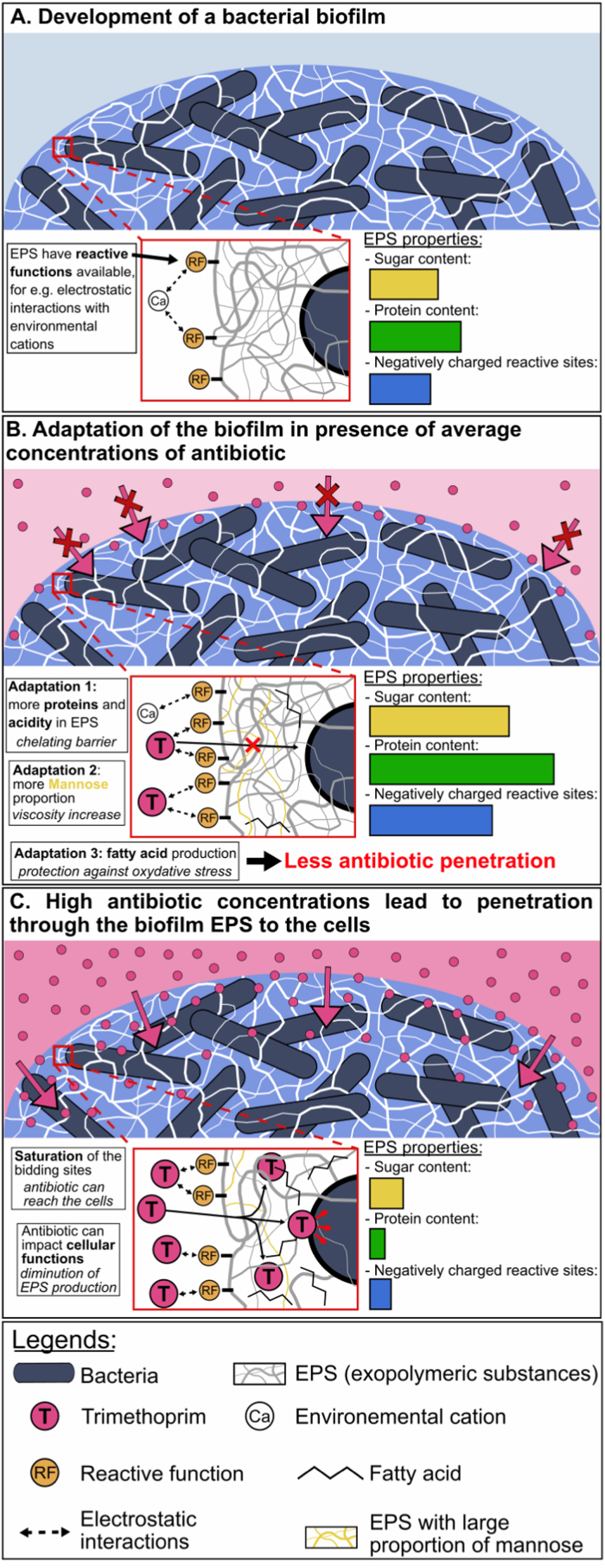
Conceptual model of the proposed adaptations in the production and chemical composition of exopolymeric substances (EPS) during exposure to antibiotics such as trimethoprim (T). (A) Bacterial biofilm with EPS containing reactive functional groups, e.g., carboxylic acids, that can form electrostatic bonds to counter ions in the surrounding environment. (B) Adaptation of EPS production and composition limiting penetration of antibiotics into the biofilm and thereby reducing their impact on the cells. The antibiotic may bind to functional groups of polysaccharides and proteins. Altered composition of polysaccharides changes viscosity and increase in fatty acid production alters the hydrophobicity of EPS as well as its antioxidant potential. (C) When the antibiotic concentration is too high, the drug saturates the biofilm's functional groups and overcomes the penetration-limiting capacity of the EPS. Thereby the effect on cells is dramatically increased.

Our bacterial strains were isolated from river biofilms and were therefore adapted to biofilm life form. Kives et al [42] found that when the same bacterial strain moved from the planktonic state to the biofilm state, it produced three times more EPS. As expected for this type of organism, our four bacterial strains were capable of secreting large amounts of EPS (Fig. 4) which could be extracted (Fig. 2). These observations are in line with the literature for the four species of this study: *Sphingomonas s.p.* [43,44], *Pseudomonas s.p.* [42,45,46], *Rhizobium s.p.* [47,48] and *Pararhizobium s.p.* [49]. Proteins were the dominant component in EPS for the four strains of this study as reported by both colorimetric assays results (Fig. 2) and can be seen in the FTIR spectra (Fig. 3). This is similar to observations done in other studies about bacterial biofilm EPS characterization [42,[50], [51], [52]]. The sugar composition, comprising mainly glucose, rhamnose, galactose and mannose, was similar to that found in the literature [43,53]. Low pH was found to significantly decrease the production of EPS in e.g., *Pseudomonas aeruginosa* [24,46], *Rhizobium tropi*ci [47] or *Lactobacillus rhamnosus GG* [54]. Our bacteria had been isolated from a relatively clean river environment and can therefore be expected to have adapted to live in an environment with neutral or slightly acidic pH (pH 6–7). This could explain the decrease in EPS amount observed in our results when bacteria were grown under acidic conditions (Fig. 2). However, results from the acid control were very different from those observed in the presence of TMP and the impact of acidity was negligible compared with the effects of TMP. A number of ways in which bacteria obtain tolerance against antibiotics have been discovered in recent years. In this study, we focused mainly on those linked to EPS in the biofilm state. Based on the results obtained, we established a conceptual model that links the modification of the physico-chemical properties of EPS to the tolerance of cells against the action of antibiotics (Fig. 7). It is interesting to note that all the strains in the study changed both the chemical composition and the quantity of EPS produced, when they came into contact with TMP even at 0.1 mM concentration (Fig. 2, Fig. 3, Fig. 5). *Pseudomonas s.p.* secreted two times more EPS when the TMP concentration reached 0.1 mM (Fig. 2). Nevertheless, this quantitative change was not sufficiently large to be visible in the SEM pictures (Fig. 4 and Table S3). The ratio between the cell and the highly hydrated EPS can be expected to be substantial in wet conditions. However, in SEM the EPS is dehydrated, collapsing the structure in various ways making quantitative estimated challenging based on SEM.

Generally, EPS production has been described to increase under antibiotic pressure or stressful conditions. This is one of the mechanisms adapted to protect cells against other microorganisms or antimicrobial agents [55]. For example, overproduction of EPS including mostly alginate had been shown to protect biofilm from antibiotics [56,57]. Hathroubi et al. [58] showed that three antibiotics, erythromycin, tetracycline, and quinpristin-dalfopristin, enhance ica gene expression in *Staphylococcus epidermidis*, which resulted in increased EPS production. A similar observation was made by Bagge et al. [21] who described that exposure to subinhibitory concentrations of the antibiotic imipenem caused an increase in biofilm volume and the induction of genes encoding alginate biosynthesis. Deletion of the locus in genes hypothesized to impact *P. aeruginosa* antibiotic resistance resulted in a two-to eight-fold reduction in biofilm-specific antibiotic resistance to tobramycin, without affecting growth or biofilm formation [59]. Interestingly, these genes are more highly expressed in biofilm than in planktonic cells, confirming the hypothesis that they play an important role in biofilm-specific antibiotic resistance.

In fact, the biofilm acts as both chemical and physical diffusion barrier to antibiotics and thus retards their penetration into the biofilm. When bacteria are dispersed from a biofilm, they usually rapidly become susceptible to antibiotics [60]. Penetration of antibiotics depends on the thickness of the biofilms, diffusion efficacy of the antibiotic, interaction of the antibiotic with the biofilm, the sorption capacity of the biofilm for the antibiotic, as well as the dose concentration and duration of the antibiotic [61]. For example, penetration of b-lactams (e.g., oxacillin, cefotaxime) and vancomycin was significantly reduced in *Staphylococcus aureus* and *Staphylococcus epidermidis* biofilms [62]. Many previous studies assume that penetration of antibiotics is limited due to the presence of reactive functions in EPS that bind antibiotics via electrostatic interactions. As the antibiotic is trapped in the matrix, it cannot reach the target cells, which are protected deeper within the biofilm. Binding of antibiotics to the EPS, thus, results in decreased antibacterial activity by a form of depletion of antibiotics [63]. For example, in *Pseudomonas aeruginosa* biofilms, polyanionic exopolysaccharides, such as alginate or Pel exopolysaccharides, are able to capture cationic antibiotics such as aminoglycosides. Thus, these polysaccharides decrease the activity of positively charged antibiotics and provide increased tolerance to these molecules [23,64,65]. Tseng et al. [19] found that the positively charged antibiotic tobramycin is sequestered in the *Pseudomonas aeruginosa* biofilm periphery, while the neutral antibiotic ciprofloxacin readily penetrated. Authors suggested that ionic interactions of tobramycin with the biofilm matrix limited its penetration. It is important to note, however, that, given enough time, tobramycin did penetrate the *P. aeruginosa* biofilms [19,66].

FTIR results for EPS extracted from the four bacterial strains, in this study, showed several reactive functional groups (e.g., carboxylic acids; Fig. 3). These can potentially interact with TMP, which is positively charged below pH 7.4 [67]. Interestingly, certain strains such as 4–19 *Rhizobium s.p.* produced an EPS with more negatively charged reactive sites (Fig. 2D) containing more proteins (Fig. 2C). The same was observed for the 4–18 *Pseudomonas s.p.* strain, although less pronounced and mainly at 0.1 mM TMP (Fig. 2C)*.* This may have been a response from the bacteria to sequester the TMP at the surface of the biofilm as the amount of EPS produced is positively correlated with colorimetric assays results (Fig. 6). Electrostatic interactions between negatively charged functional groups in the EPS would prevent positively charged molecules from coming into contact with the cells. This mechanism, linked with special types of polysaccharides (Psl and Pel), are suspected to provide antibiotic resistance in e.g., *P. aeruginosa* strains (Colvin et al., 2011; [18]). The increase in negatively charged reactive sites may also reveal changes in surface charge, that may repel TMP and prevent it from reaching the cells. Similar observations were made by Pagès et al. [68] with bacterial cells secreting negatively charged lipopolysaccharides (LPS) to provide a repulsion barrier to the diffusion of the hydrophilic β lactams into the cell.

EPS contains a mixture of proteins and it is interesting to note that in the FTIR spectra, the ratio of the amide I (∼1650 cm^−1^; band III; Fig. 3) and amide II (∼1550 cm^−1^; band IV; Fig. 3) band intensities vary considerably from one species to another, but also depending on the treatment. Amide I and amide II correspond to different parts of the peptide bond in proteins. Analysis of the secondary structure (e.g., α-helix or β-sheet) of proteins is done nearly exclusively by the amide I band [69]. The difference in ratios of amide I and amide II bands can in our case mean either a different secondary structure of the same proteins, or the presence of different proteins altogether. Since EPS is a complex mixture of macromolecules, the FTIR spectra are plagued by significant overlaps (e.g., bands of proteins and fatty acids), making it impossible to reliably decompose the spectrum. Thus, although the changes are noticeable, they are inconclusive as to whether the amide I and II band ratio change originates from a change in protein organization or from the production of new proteins to adapt to the presence of antibiotics, or both. On possible cause of this change could be the secretion of stress-response enzymes in the EPS matrix. This was demonstrated by Bagge et al. [70] for *P. aeruginosa* and Anderl et al. [71] for *K. pneumoniae* where biofilm matrix accumulates β-lactamases to hydrolyse antibiotics. Bowler et al. [72] discovered the same mechanism in mature *P. aeruginosa* biofilms, which are more resistant to ceftazidime due to an increased amount of β-lactamase in the matrix compared to younger biofilms. However, our FTIR spectroscopic data is inconclusive in this regard and thus we are unable verify this hypothesis in our case. Nevertheless, the results of the physico-chemical characterization of EPS (Fig. 2) are in line with strain-adaptation in the production and chemical composition of EPS in response to antibiotics. In other words, in contact with TMP, bacteria appeared to adapt their EPS production and EPS chemistry to increase the content of proteins and reactive groups (*adaptation 1*; Fig. 7B).

Different mathematical models have shown that in addition to interaction and sorption, delayed antibiotic penetration can be affected by biofilm thickness [73]. The EPS sugar composition analysis showed that, already at 0.1 mM, the presence of TMP led to a significant increase in the proportion of mannose for all the bacterial strains (Fig. 5C). Previous work has shown that bacterial EPS with a high proportion of mannose give rise to EPS with high viscosity and water retention [74]. The increase in the proportion of mannose in the EPS of our strains could therefore indicate a more viscous EPS with limited penetration of TMP, which would be beneficial for the bacterial cells. This mechanism is presented in the conceptual model as a second change in the EPS matrix in response to antibiotics (*adaptation 2*; Fig. 7B).

A more detailed study of EPS chemistry revealed another change following TMP exposure. Change in EPS chemistry could be linked to a greater quantity of fatty acids [75]. When the concentration of TMP reached a certain level (1 mM for 4–6 *Sphingomonas s.p.*, 4–18 *Pseudomonas s.p* and 4–19 *Rhizobium s.p.* and 5 mM for 4–30 *Pararhizobium s.p.*) FTIR spectra displayed a higher proportion of fatty acids (Fig. 3). TMP is well known to induce oxidative stress in organisms [76,77] and fatty acids may be a response to this stress [[78], [79], [80]]. A similar observation was reported by Van Acker et al. [81] where genes encoding products with antioxidant properties were upregulated in biofilms formed by *Burkholderia cepacian* following tobramycin treatment. It is possible that following TMP exposure, bacteria secreted fatty acids and/or lipids in EPS matrix to reduce its permeability and change its physico-chemical characteristics. Our dataset does not allow us to conclude what type of fatty acid may increase. However, a change in fatty acid composition may arise due to membrane restructuring, increased formation and release of membrane vesicles or increased production of individual fatty acids not forming part of larger biomolecules [[82], [83], [84]]. This mechanism, which may alter the hydrophobicity of the EPS matrix, is presented in the conceptual model as a third change in the biofilm in response to antibiotics (*adaptation 3*; Fig. 7B). Same observations have been made by Uzoechi & Abu-Lail [85] with *E. coli* increased its biofilm hydrophobicity upon exposure to ampicillin.

In short, the observed changes in EPS quantity and chemistry are expected to have altered the penetration of antibiotics. This may have provided time for other adaptive phenotypic responses, which could reduce susceptibility further [19]. It has been proposed that antibiotics that penetrate more slowly through biofilm EPS may give time for an adaptive phenotypic response that could increase tolerance [19,66] or inactivation or degradation of antimicrobials by enzymes present in biofilm matrix [17]. Our methods of analysis do not allow us to answer if this took place in our system. Future studies, for example using gene expression linked to TMP exposure could possibly shed light on such time-resolved processes.

The collected data show that when the TMP concentration reached a threshold, the quantity and chemical composition of the EPS dramatically changed for all strains (Fig. 2, Fig. 5). This was probably due to cellular response to the high antibiotic concentration inhibiting cellular functions and could explain why EPS properties and TMP are anticorrelated (Fig. 6). This effect is particularly pronounced for strains 4–19 and 4–30, for which four times less EPS is produced at a concentration of 5 mM of TMP (Fig. 2; Table 2). Similar effects have been shown, for example for *B. subtilis*, *E. coli* and *P. aeruginosa* during antibiotic exposure [21,86]. Functional groups in the EPS may have become saturated and the penetration of the antibiotic into the biofilm therefore increased. As a result, the bacterial cells became too affected to maintain their cellular functions and secrete EPS. For each strain, there appears to have been an individual TMP threshold, above which the bacteria no longer could produce enough EPS to protect themselves and stop the antibiotic penetrating through the biofilm. Above this threshold, cells decreased due to the action of the antibiotic, which further limits the production of EPS. This mechanism could explain the observed decrease in the quantity of EPS produced by cells that were exposed to higher concentrations than 1 mM TMP (Fig. 2, Fig. 5). This hypothesis is shown in the last step of the conceptual model (Fig. 7C).

**Table 2:** EPS production of each strain linked with trimethoprim quantity. The percentage is based on amount produced without Trimethoprim addition.

Strains	0.1 mM TMP	1 mM TMP	5 mM TMP
4_6	93 %	160 %	165 %
4_18	248 %	124 %	101 %
4_19	52 %	103 %	22 %
4_30	111 %	98 %	26 %

## 5 Conclusion

Four river bacterial strains capable of forming biofilms through the secretion of exopolymeric substances (EPS) were exposed to increasing concentrations of trimethoprim. The EPS was extracted, purified and characterized using colorimetric tests, FTIR and GC-MS. Morphological characterization was also carried out using SEM. The results showed that EPS quantity and chemistry EPS changed for all strains starting at a concentration of 0.1 mM trimethoprim. The combined results suggest that environmental bacterial strains adapt their EPS production and the chemical composition of EPS in response to antibiotic exposure. We hypothesize that bacteria benefit from the change in EPS chemistry since it limits the penetration of trimethoprim into the biofilm and thus protects the cells from the action of the antibiotic. Three main mechanisms are proposed: (i) an increase in the proportion of proteins and reactive functional groups in the biofilm matrix that can bind antibiotics, (ii) an increase in the proportion of mannose to make the biofilm more viscous and (iii) a change in EPS chemistry giving an increase in the proportion of fatty acids, protecting cells against antibiotic-related oxidative stress. Our results also suggest that above a threshold, the antibiotic concentration became too high for the protective environment of the cells. Once the antibiotics reached the cells, their ability to produce EPS and maintain other cellular functions became severely impaired. This can explain the sharp decrease in the quantity of secreted EPS at the highest trimethoprim concentrations. Finally, the different strains in our study presented different antibiotic responses, probably linked to their levels of antibiotic tolerance. To conclude, this study shows that EPS represents a key factor in antibiotic tolerance of bacteria via multiple mechanisms. Thus, this study broadens the discussion concerning antibiotic-resistance as it presents additional processes that may work in tune with genetically acquired resistance to enhance bacterial tolerance to antibiotics. These processes are of importance both for maintaining healthy ecosystems during pharmaceutical pollution, as well as for targeting diseases caused by pathogenic bacteria in health care systems.

## Declaration of competing interest

The authors declare the following financial interests/personal relationships which may be considered as potential competing interests: Madeleine Ramstedt reports financial support was provided by Kempe-Carlgrenska Foundation Fund. If there are other authors, they declare that they have no known competing financial interests or personal relationships that could have appeared to influence the work reported in this paper.

## CRediT authorship contribution statement

**Thibault Duteil:** Writing – original draft, Methodology, Investigation, Formal analysis, Data curation, Conceptualization. **Andras Gorzsás:** Writing – review & editing, Supervision. **Madeleine Ramstedt:** Writing – review & editing, Supervision, Project administration, Funding acquisition.

## Data Availability

Data will be made available on request.
