# Supplementary material for: Quantitative and chemical adaptation of exopolymeric substances formed by a river microbial consortium during exposure to the antibiotic trimethoprim
Source: Biofilm. 2025 Nov 19;10:100334. doi: 10.1016/j.bioflm.2025.100334 (PMC12677181; doi:10.1016/j.bioflm.2025.100334)
Supplement: Multimedia component 1 [file mmc1.docx]

| **Strain** | **Medium** | **Weight** | **Sugar** | **Protein** | **Alcian** | **Glu** | **Rha** | **Man** | **Gal** | **Xyl** | **Fuc** | **Ara** |
| --- | --- | --- | --- | --- | --- | --- | --- | --- | --- | --- | --- | --- |
|  | mM Tri | µg | mg eq. Xan/mL EPS | mg eq. Alb/mL EPS | mg eq. Xan/mL EPS | µg/mg DW | µg/mg DW | µg/mg DW | µg/mg DW | µg/mg DW | µg/mg DW | µg/mg DW |
| 4_6 | 0 |  | 1,1E-02 | 3,3E+02 | 1,9E-02 | 1,0E+01 | 5,3E+00 | 8,9E-02 | 1,0E+00 | 2,9E-01 | 9,1E-02 | 6,4E-02 |
| 4_6 | 0 | 633 | 1,1E-02 | 3,2E+02 | 1,7E-02 | 8,2E+01 | 3,1E+01 | 4,4E-02 | 4,1E+00 | 3,0E+00 | 7,3E-01 | 7,0E-01 |
| 4_6 | 0 |  | 8,1E-03 | 3,3E+02 | 2,1E-02 | N.A | N.A | N.A | N.A | N.A | N.A | N.A |
| 4_6 | 0,1 |  | 9,8E-03 | 1,4E+02 | 1,3E-02 | 1,6E+01 | 7,2E+00 | 4,0E+00 | 1,9E+00 | 3,9E-01 | 9,4E-02 | 5,5E-02 |
| 4_6 | 0,1 | 590 | 7,2E-03 | 1,3E+02 | 1,2E-02 | 1,2E+01 | 6,3E+00 | 3,8E+00 | 2,0E+00 | 6,1E-01 | 4,4E-01 | 4,3E-01 |
| 4_6 | 0,1 |  | 7,6E-03 | 1,3E+02 | 1,3E-02 | N.A | N.A | N.A | N.A | N.A | N.A | N.A |
| 4_6 | 1 |  | 8,6E-03 | 4,7E+01 | 7,5E-03 | 3,5E+01 | 1,0E+01 | 6,4E+00 | 1,8E+00 | 1,6E+00 | 1,2E-01 | 1,4E-01 |
| 4_6 | 1 | 1015 | 8,2E-03 | 5,8E+01 | 9,0E-03 | 4,7E+00 | 2,4E+00 | 1,0E+00 | 4,3E-01 | 2,0E-01 | 6,4E-02 | 5,0E-02 |
| 4_6 | 1 |  | 5,6E-03 | 4,6E+01 | 1,3E-02 | N.A | N.A | N.A | N.A | N.A | N.A | N.A |
| 4_6 | 5 |  | 6,8E-03 | 4,8E+01 | 7,2E-03 | 2,0E+01 | 9,3E+00 | 4,2E+00 | 1,1E+00 | 4,0E-01 | 1,3E-01 | 8,9E-02 |
| 4_6 | 5 | 1044 | 4,3E-03 | 4,9E+01 | 5,8E-03 | 2,2E+01 | 9,6E+00 | 4,8E+00 | 1,2E+00 | 6,0E-01 | 1,5E-01 | 1,5E-01 |
| 4_6 | 5 |  | 4,6E-03 | 4,4E+01 | 7,8E-03 | N.A | N.A | N.A | N.A | N.A | N.A | N.A |
| 4_6 | acid |  | 4,6E-03 | 1,0E+02 | 8,9E-03 | 6,6E+00 | 3,6E+00 | 1,7E+00 | 4,5E-01 | 3,6E-01 | 2,5E-01 | 2,7E-01 |
| 4_6 | acid | 220 | 4,5E-03 | 9,8E+01 | 9,3E-03 | N.A | N.A | N.A | N.A | N.A | N.A | N.A |
| 4_6 | acid |  | 4,6E-03 | 9,7E+01 | 9,1E-03 | N.A | N.A | N.A | N.A | N.A | N.A | N.A |
| 4_18 | 0 |  | 5,8E-03 | 2,3E+02 | 1,5E-02 | 1,1E+01 | 1,8E+00 | 6,8E-02 | 1,4E-01 | 3,9E-01 | 9,4E-02 | 9,7E-02 |
| 4_18 | 0 | 891 | 6,7E-03 | 2,1E+02 | 1,5E-02 | 1,1E+01 | 1,3E+00 | 8,8E-02 | 1,5E-01 | 4,1E-01 | 1,3E-01 | 8,7E-02 |
| 4_18 | 0 |  | 8,1E-03 | 2,2E+02 | 1,4E-02 | N.A | N.A | N.A | N.A | N.A | N.A | N.A |
| 4_18 | 0,1 |  | 7,1E-03 | 2,5E+02 | 2,1E-02 | 3,5E+01 | 4,4E+00 | 4,7E-01 | 2,7E-01 | 2,6E-01 | 1,2E-01 | 1,1E-01 |
| 4_18 | 0,1 | 2207 | 7,3E-03 | 2,5E+02 | 2,2E-02 | 3,5E+01 | 4,4E+00 | 3,8E-01 | 2,2E-01 | 2,8E-01 | 8,5E-02 | 7,6E-02 |
| 4_18 | 0,1 |  | 7,5E-03 | 2,4E+02 | 2,1E-02 | 2,8E+01 | 5,7E+00 | 3,0E-01 | 2,2E-01 | 1,9E-01 | 1,1E-01 | 1,8E-01 |
| 4_18 | 1 |  | N.A | 1,4E+02 | 1,5E-02 | 2,5E+01 | 2,3E+00 | 1,5E+00 | 5,1E-01 | 1,0E+00 | 9,7E-02 | 1,3E-01 |
| 4_18 | 1 | 1103 | 5,9E-03 | 1,5E+02 | 1,4E-02 | 1,7E+01 | 1,6E+00 | 1,1E+00 | 4,0E-01 | 6,8E-01 | 7,3E-02 | 1,1E-01 |
| 4_18 | 1 |  | 7,7E-03 | 1,4E+02 | 1,1E-02 | N.A | N.A | N.A | N.A | N.A | N.A | N.A |
| 4_18 | 5 |  | 2,5E-03 | 4,7E+01 | 1,6E-03 | 8,1E+00 | 1,1E+00 | 3,5E-01 | 5,4E-02 | 2,9E-01 | 8,0E-02 | 9,7E-02 |
| 4_18 | 5 | 901 | 2,6E-03 | 3,7E+01 | 8,5E-03 | 1,8E+01 | 2,6E+00 | 6,3E-01 | 1,5E-01 | 4,5E-01 | 1,3E-01 | 1,2E-01 |
| 4_18 | 5 |  | 4,5E-03 | 4,7E+01 | 1,1E-02 | N.A | N.A | N.A | N.A | N.A | N.A | N.A |
| 4_18 | acid |  | 3,7E-03 | 5,9E+01 | -1,7E-04 | 6,1E+00 | 5,0E-01 | 6,3E-01 | 6,8E-01 | 4,9E-01 | 3,0E-01 | 3,6E-01 |
| 4_18 | acid | 160 | 2,3E-03 | 5,2E+01 | 6,6E-04 | N.A | N.A | N.A | N.A | N.A | N.A | N.A |
| 4_18 | acid |  | 3,7E-03 | 6,3E+01 | 8,9E-03 | N.A | N.A | N.A | N.A | N.A | N.A | N.A |

**Table S1:** Numerical data used in the study for strains 4-6 and 4-18.
