# Supplementary material for: Quantitative and chemical adaptation of exopolymeric substances formed by a river microbial consortium during exposure to the antibiotic trimethoprim
Source: Biofilm. 2025 Nov 19;10:100334. doi: 10.1016/j.bioflm.2025.100334 (PMC12677181; doi:10.1016/j.bioflm.2025.100334)
Supplement: Multimedia component 3 [file mmc3.docx]

|  | **no TMP** |  | **1 mM TMP** |  |
| --- | --- | --- | --- | --- |
| **Strains** | **cells surface (µm^2^)** | **EPS coverage (%)** | **cells surface (µm^2^)** | **EPS coverage (%)** |
| 4_6 | 0.31±0.06 | 27±21 | 0.49±0.08 | 41±20 |
| 4_18 | 0.33±0.09 | 13±8 | 0.54±0.06 | 26±12 |
| 4_19 | 0.61±0.13 | 45±18 | 0.65±0.13 | 31±20 |
| 4_30 | 0.49±0.15 | 39±28 | 0.43±0.07 | 41±24 |

**Table S3:** Average results of cells surface measures and EPS coverage area from SEM pictures based on ten cells randomly selected.
